# Trends and patterns of suicidal behaviour in Nigeria: Mixed-methods analysis of media reports from 2016 to 2019

**DOI:** 10.4102/sajpsychiatry.v27i0.1572

**Published:** 2021-03-08

**Authors:** Olushola Olibamoyo, Bolanle Ola, Olurotimi Coker, Abiodun Adewuya, Akintayo Onabola

**Affiliations:** 1 Department of Behavioural Medicine, Faculty of Clinical Sciences, Lagos State University College of Medicine, Lagos, Nigeria; 2 Department of Medicine, Oaks Hospitals, Lagos, Nigeria

**Keywords:** content analysis, media, trend, patterns, suicidal behaviours, Nigeria

## Abstract

**Background:**

Media coverage of suicides in Nigeria appears to be explicitly descriptive and deviates from the recommended best practice. Despite these shortcomings, verifiable information provided by these media outlets could arguably, at the minimum, mirror the reality of the trends and patterns of suicidal behaviour in Nigeria.

**Aim:**

This study aimed to analyse the trends and patterns of suicidal behaviour in Nigeria using media reports from 2016 to 2019. We examined the effect of gender and age groups on these trends and patterns of suicidal behaviour.

**Setting:**

The study was carried out in Nigeria.

**Methods:**

Qualitative content analysis was used to assess the content of each verifiable suicide event. In total, 336 verified suicide-related events were selected from 4365 media reports. Quantitative data were collected on age, gender, type of suicidal behaviour, method, place and motivation for suicidal behaviour. Data were analysed using the Statistical Package for the Social Sciences software. Fisher’s exact test was used to examine the association between gender, age groups and other variables. *p*-value was set at ≤ 0.05.

**Results:**

Completed suicide was the most common reported suicidal behaviour. Hanging was the dominant reported method, followed by poisoning. Significant gender differences were observed between age groups (*p* < 0.001) and methods of suicidal behaviour (*p* < 0.001). Also, significant age differences were observed between the methods of suicidal behaviour (*p* < 0.001), places (*p* < 0.001) and motivations for suicidal behaviour (*p* < 0.001).

**Conclusion:**

The study confirms that there are gender and age differences in the trends and patterns of suicidal behaviour in Nigeria.

## Introduction

Suicidal behaviour is a leading cause of injury and death worldwide. It comprises suicide ideation (thoughts of engaging in behaviour intended to end one’s life), suicide planning (formulation of a specific method through which one intends to die), suicide attempt (engagement in self-injurious behaviour in which there is at least some intent to die) and suicide.^1^ According to the World Health Organization (WHO), 75.5% of suicides in the world occurs in low- and middle-income countries, yet they have contributed only 8% of information about suicide.^1^ To address the paucity of information amongst low- and middle-income countries, which include Africa with a suicide rate of 12.0/100 000 population (which is higher than the world rate of 10.5/100 000 population),^2^ the WHO called for action on reducing sociocultural stigmatisation of suicidal behaviour, decriminalisation of suicidal behaviour and standardisation of deaths by suicide through compulsory registration of deaths amongst their population.^3^ Amongst African countries, the rate of suicide in Nigeria of 17.3/100 000 population is among the highest.^4^

Across Nigeria, research into suicide started in the 1960s when its incidence began to be formally recorded.^4^

Undoubtedly, trends and patterns of suicidal behaviour are important in having a clear understanding of the size of the problem, its distribution in the population and the major risk and protective factors at individual and community levels. However, most of the studies on suicide in Nigeria have been on ideation and occasionally on attempts without clear data on the trends and patterns of suicide itself.^5,6,7,8^ The dearth of studies on the trends and patterns of suicide in Nigeria may be because of the lack of reliable health information, poor death registration and criminalisation of suicidal behaviour in Nigeria.^3^ Unfortunately, ample information exists in news media reports of suicide to overcome the hurdle of a lack of information about suicidal behaviour.

However, researchers have highlighted the gap between the epidemiological data of suicide in the population and news media reports of suicide.^9^ Nonetheless, it is useful to analyse verifiable information (albeit crude) about suicidal behaviour, provided by media outlets, which could arguably, at the minimum, mirror the reality of the trends and patterns of suicidal behaviour in settings such as Nigeria where there are low resources as well as the paucity of funded research.

Apparently, given the 200 million population of the country,^10^ Nigeria’s media scene is one of the liveliest in Africa.^11^ Each of the 36 states runs at least one radio network and a television (TV) station. There are more than 100 national and local press titles.^11^ However, today in Nigeria, the digital landscape has re-invented how news consumers access news, share news and push news, causing the previously vibrant traditional media to have online platforms where they could offer stronger online news contents^12^ – leading to fierce competition, increased sensationalism of reports and lowering the bar about which information is newsworthy.^12^ Therefore, news media coverage of suicidal behaviour in Nigeria appears to be sensational, explicitly descriptive and deviates from recommended best practice. Also, there is an abundance of evidence that certain types of media coverage of suicide can increase the likelihood of recurrence of suicidal behaviour (Werther effect).^13,14,15,16^

In this study, we used data provided by media reports of suicidal behaviour to analyse the trends and patterns of suicidal behaviour in Nigeria from 2016 to 2019. We examined the effects of gender and age groups on these trends and patterns of media-reported suicidal behaviour.

## Methods

We followed the guidelines for Good Reporting of a Mixed Methods Study which comprises justification for using a mixed-methods approach, description of design, description of each method, description of where the integration has occurred, any limitations of one method associated with the presence of another method and any insights gained from mixing methods.^17^ The mixed-method approach used in the study helped to triangulate the data and provided a more robust analysis. We quantified the number of suicidal behaviours published in media reports and verifiable suicidal behaviour from 01 January 2016 to 31 December 2019.

However, quantifying the number of verifiable suicidal behaviour by itself is weak in understanding the context, content, trends and patterns of the reported articles, something that the qualitative content analysis evaluated. On the other hand, qualitative content analysis holds the potential for biased interpretations, and quantitative research does not have this weakness. So we quantified the frequency of each code for quantitative analysis.

### News media selection

Given that about 122.7 million Nigerians (61.4% of the population) have access to the Internet,^18^ and the Internet is the most rapidly growing method of accessing news media particularly for middle-aged and young adults in Nigeria,^12^ we looked at the top five Internet-based media sources for news used by Nigerians as determined and ranked by Alexa.com, an online tool ranking websites, based on multiple usage indicators.^19^ They are as follows: three print media with online platform – www.punchng.com (17.37 million visits in the last 6 months), www.thenationonlineng.net (7.86 million visits in the last 6 months) and www.dailypost.ng (7.4 million visits in the last 6 months) – and two news blogs www.nairaland.com (33.86 million visits in the last 6 months) and www.lindaikejisblog.com (9.14 million visits in the last 6 months).

### Search strategy

**FIGURE 1:**
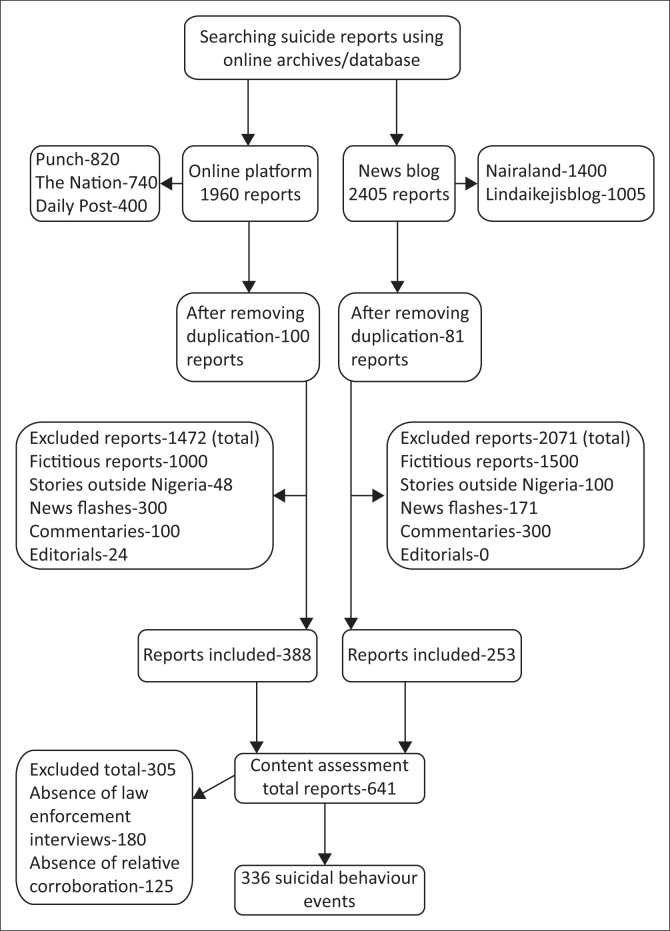
Selection process of media-reported suicide events from 2016 to 2019.

To assess the relevant media reports on suicidal behaviour published on five online platforms from 01 January 2016 to 31 December 2019, Google and Bing (common search engines used in Nigeria) were searched using the following keywords, namely, one core word (suicide), two synonyms (self-murder and self-destruction) and two hyponyms (kill self and take life) of suicide. We followed the principles of the Preferred Reporting Items for Systematic Reviews and Meta-Analyses (PRISMA) statement.^20^ The results of the identification, screening, eligibility assessment and inclusion of the media articles are presented in Figure 1. We left out news published before this period because of missing online reports.

### Sampling strategy

We used a purposive sampling technique. The authors acknowledge that despite an extensive search, few articles may have been omitted. A total number of 4365 media reports were reviewed. After removing the duplicates, the number of media reports included in the screening process was 4184. Two of the authors (O.O. and B.O.) were involved in the screening of reports. In total, 3543 non-relevant media reports, based on the exclusion criteria, were excluded and the remaining 641 reports were assessed for eligibility based on the inclusion criteria by another author (O.C.). Ultimately, 336 suicidal behaviour events were identified.

Another author (A.A.) performed the final quality check. The selected 336 suicidal behaviour events were based on the following inclusion criteria:

1. Publications in Nigeria about suicide events that took place in Nigeria.
2. The suicide events were defined as suicide deaths, attempted suicides, homicide suicide and suicidal ideation.
3. Suicide events were termed verifiable for the study only when they meet all the following criteria:
  - At least two news media outlets reported the particular suicide event.
  - Evidence of corroboration of the particular suicide event by law-enforcement agents.
  - Evidence of interview with relatives, friends or neighbours.

The two most common reasons for exclusion were that they did not have any supporting interviews either with the law-enforcement agents or with relatives or neighbours.

### Qualitative analysis

To determine the dominant trends and patterns of suicidal behaviour in Nigeria, in terms of age, gender, type of suicidal behaviour, methods used, place and narratives of motivation, two authors (O.O. and B.O.) manually noted many plausible initial open codes across the whole data set. These open codes were both data and theory driven. The second stage focused on the research team reading the materials closely and agreeing on a set of initial preliminary codes that were considered relevant to the data to produce a manageable list of headings that would account for all the data in the material. The third stage was to incorporate the initial open codes with similar preliminary codes to aid the search for categories and emerging themes. The fourth stage involved further review and refining of the themes and selected extracts that supported and described the themes. These codes were then thoroughly discussed and agreed on by consensus by members of the research team to reduce bias and increase the credibility as well as the trustworthiness of the findings through cross validation. The codebook is presented in Table 1.

**TABLE 1:** Codebook for the abstracted data.

Variable	Code	Theme/text
Identity	Suicide victim Suicide survivor	A person who died by suicide A person who has survived any suicidal crisis
Type of suicidal behaviour	Completed suicide Attempted suicide Homicide suicide Suicidal ideation	Item is on a case of completed suicide Item is on a case of attempted suicide or a suicidal act stopped by another person immediately before the act Item is on a homicide (attempt); murderer is suicidal Item is on suicidal ideation not accompanied by suicidal attempt or suicide. Also, self-aborted suicide attempt in which an individual is one step away from attempting suicide but changes her or his mind
Method of suicide	Hanging Jumping Firearm Self-inflicted injury Poisoning Under vehicle/train Fire/self-immolation Others (diving, drowning)	Hanged himself or herself Jumped to death from a height Shot self to death Slit throat, stab self, slash wrist to death Self-poisoned by drinking pesticide, acid, weedicide and other poisonous chemicals Death by standing in front of a moving vehicle or train Death by setting fire on self directly or indirectly on properties within the same vicinity Death by drowning in a river or diving into a sea
Place where the suicidal behaviour occurred	Home Workplace/school Place of worship/hospitals Notable public sites (bridges, buildings) Farm/forest/local river Other (court, police cell)	Place of residence of the victim/survivor Place, where he/she works or attend school Religious house or medical centre Public structures or buildings, for example, bridges, malls etc. Farms, bushes, local rivers and forests Courtroom or premises, detention centres and correctional homes
Motivation for suicidal behaviour refers to both assigned (suggested by police, relatives and friends in the article) and confessed (suicide notes, confessions before death and after attempted suicide) reasons given for the suicidal behaviour	Relationship difficulties Financial difficulties Poor academic record Job difficulties Mental illness Loss events Multiple causes Others	Family dispute, spousal/partner infidelity and domestic abuse or violence Failure to meet financial obligation to self and family Exam failure or unsatisfactory grades Loss of job or inability to secure satisfactory employment or unsatisfactory work environment Being withdrawn, odd behaviours, sudden change in behaviour and severe agitation Loss of money, properties and reputation from gambling, fraud and false accusation More than one cause Illnesses, childlessness
Intention of suicidal behaviour	Verbal threats Observable change in behaviour Previous attempt	Verbally expressed intention to kill self, hurt self or surprise relatives before suicide event Observable change in behaviour which is different from his or her expected behaviour such as keeping to self, change in mood and agitation Previous attempts at killing self or hurting self
Age groups in years	Less than 18 18–29 30–44 45–59 60 and above	As reported
Gender	Male Female	As reported
Suicide note	Present Absent	As reported
Prosecution by law-enforcement agents	Yes No	As reported

### Quantitative analysis

The quantitative analysis aimed at measuring associations between item content and suicidal behaviour rates. Data were coded and entered into the Statistical Package for the Social Sciences^21^ (SPSS) version 23.0 and cleaned. Categorical variables were summarised with frequencies and percentages. Fisher’s exact tests examined the association between gender and age, type of suicidal behaviour, place and motivation for suicidal behaviour. *p-*value was set at ≤ 0.05.

### Ethical consideration

This retrospective review qualifies for ‘exempt’ status under human subjects’ regulations, given that it involved the use of existing records of past media reports and that no items of data collected that enabled the identification of any subject recorded and the data are not linked to subject identifiers.

## Results

Three hundred and thirty-six verifiable suicidal behaviours were retrieved and included in the analysis from the 641 media reports of suicide from the top five news media in Nigeria. The most common (78.9%) of the suicidal behaviours was completed suicide, 30.1% of the persons reported were between 18 and 29 years of age and the range was 8–84 years. Victims and/or survivors were predominantly men (77.4%), hanging (41.1%) was the most common reported method and 77.5% of the suicide events took place at home.

Relationship difficulties (24.4%) were the most reported narratives for motivation of the suicidal behaviour, and verbal threats to harm self or die by suicide were the most common reported sign of intention. Suicide notes were left in 17.9% of the suicide events and 4.5% were reportedly charged and prosecuted at the courts by law-enforcement agents. The rest of the results are presented in Table 2.

**TABLE 2:** Characteristics of suicide events as reported by media reports of Nigerian news media from January 2016 to December 2019.

Variables	No. of observed suicide events *N* = 336	%
**Type of suicidal behaviour**		
Completed suicide	265	78.9
Attempted suicide	48	14.3
Homicide suicide	22	6.5
Suicide ideation	1	0.3
**Year**		
2016	59	17.6
2017	90	26.8
2018	78	23.2
2019	109	32.4
**Age group (in years)**		
Less than 18	28	8.3
18–29	101	30.1
30–44	72	21.4
45–59	41	12.2
60 and above	15	4.5
Unidentified	79	23.5
Range from identifiable age reported: 8–84 years		
**Gender**		
Male	260	77.4
Female	76	22.6
**Reported method of suicide**		
Hanging	138	41.1
Jumping	38	11.3
Firearm	11	3.3
Self-inflicted injury	13	3.9
Poisoning	115	34.2
Under vehicle/train	2	0.6
Fire/self-immolation	5	1.5
Diving/drowning	4	1.2
Unidentified	10	3.0
**Place where the event took place**		
Home	237	70.5
Workplace/school	19	5.7
Place of worship/hospitals	8	2.4
Notable public sites	39	11.6
Farm/forest/local river	25	7.4
Court/police cell	5	1.5
Unidentified	3	0.9
**Narratives of motivation**		
Relationship difficulties	82	24.4
Financial difficulties	55	16.4
Poor academic record	18	5.4
Job difficulties	17	5.1
Mental illness	58	17.3
Loss events	24	7.1
Physical illness/childlessness	12	3.6
Multiple causes	2	0.6
Unidentified	68	20.2
**Signs of intention**		
Verbal threats	82	24.4
Observable change in behaviour	49	14.6
Previous attempt	20	6.0
Unidentified	185	55.1
**Reported suicide notes**		
No	276	82.1
Yes	60	17.9
**Arrested and prosecuted by law enforcement**		
No	321	95.5
Yes	15	4.5
Completed suicide (relatives)	2	-
Homicide suicide	1	-
Attempted suicide	12	-

*N*, total frequency; (%), percentage.

Unidentified, information not available in the reviewed media report.

### Gender differences on age groups of reported persons, type of suicidal behaviour, reported methods, places and motivations for suicidal behaviour

Significant differences (*p* < 0.001) were noted between gender and age group; 22.4% of all women as against 4.2% of all men fell into the less than 18 years’ age group, whilst 42.1% of all women as against of 26.5% of all men were aged 18–29 years. However, the trend was reversed from 30 years and above. Conversely, women represented 60.7% of victims and/or survivors under 18 years age but the trend was reversed in other age groups that were predominantly men.

Significant association (*p* < 0.001) was observed between gender and reported methods of suicide events. In women, poisoning (64.5%), hanging (18.4%) and jumping (6.6%) were the most common methods, whereas, in men it was hanging (47.7%), poisoning (25.4%) and jumping (12.7%). The rest of the results are presented in Table 3 and Table 4.

**TABLE 3:** Gender and type of suicide events, age groups, reported method, place and motivation.

Variables	Gender				Fisher’s exact test (*p*)
	Female no. observed		Male no. observed		
	*N* = 76	%	*N* = 260	%	
**Type**					
Completed suicide	60	78.9	205	78.8	0.278
Attempted suicide	14	18.4	34	13.1	
Homicide suicide	2	2.6	20	7.7	
Suicide ideation	0	0.0	1	0.4	
**Age group (years)**					
Less than 18	17	22.4	11	4.2	**< 0.001**
18–29	32	42.1	69	26.5	
30–44	9	11.8	63	24.2	
45–59	5	6.6	36	13.8	
Above 60	2	2.6	13	5.0	
Unidentified	11	14.5	68	26.2	
**Method of suicide**					
Hanging	14	18.4	124	47.7	**< 0.001**
Jumping	5	6.6	33	12.7	
Firearm	0	0.0	11	4.2	
Self-inflicted injury	2	2.6	11	4.2	
Poisoning	49	64.5	66	25.4	
Under vehicle/train	0	0.0	2	0.8	
Fire/self-immolation	1	1.3	4	1.5	
Diving/drowning	1	1.3	3	1.2	
Unidentified	4	5.3	6	2.3	
**Place**					
Home	61	80.3	176	67.7	0.067
Work/school	6	7.9	13	5.0	
Worship/hospital	0	0.0	8	3.1	
Notable public sites	3	3.9	36	13.8	
Farm/forest/local river	4	5.3	21	8.1	
Court/police cell	1	1.3	4	1.5	
Unidentified	1	1.3	2	0.8	
**Motivation**					
Relationship difficulties	32	42.1	50	19.2	0.81
Financial difficulties	6	7.9	49	18.8	
Poor academics	6	7.9	12	4.6	
Job difficulties	1	1.3	16	6.2	
Mental illness	12	15.8	46	17.7	
Loss events	7	9.2	17	6.5	
Illness/childlessness	3	3.9	9	3.5	
Multiple causes	0	0.0	2	0.8	
Unidentified	9	11.8	59	22.7	

*N*, total frequency; %, percentage; *p*, level of significance.

Unidentified, information not available in the reviewed media report.

**TABLE 4:** Age group and type of suicide events, gender, method, place and motivation.

Variables	Age groups (years)												Fisher’s exact test (*p*)
	< 18		18–29		30–44		45–59		≥ 60		Unidentified		
	*N*	%	*N*	%	*N*	%	*N*	%	*N*	%	*N*	%	
**Type**													
Completed suicide	25	89.3	82	81.2	54	75.0	34	82.9	14	93.3	56	70.9	0.09
Attempted suicide	3	10.7	16	15.8	10	13.9	5	21.2	1	6.7	14	17.7	
Homicide suicide	0	0.0	3	3.0	7	9.7	2	4.9	0	0.0	9	11.4	
Suicidal ideation	0	0.0	0	0.0	1	1.4	0	0.0	0	0.0	0	0.0	
**Gender**													
Female	17	60.7	32	31.7	9	12.5	5	12.2	2	13.3	11	13.9	**< 0.001**
Male	11	39.3	69	69.3	63	87.5	36	87.8	13	86.7	68	86.1	
**Method**													
Hanging	11	39.3	31	30.7	33	48.5	23	56.1	11	73.3	29	36.7	**< 0.001**
Jumping	1	3.6	8	7.9	9	12.5	7	17.1	1	6.7	12	15.2	
Firearm	0	0.0	0	0.0	2	2.8	0	0.0	2	13.3	7	8.9	
Self-inflicted injury	0	0.0	5	5.0	3	4.2	3	7.3	0	0.0	2	2.5	
Poisoning	15	53.3	49	48.5	21	29.2	8	19.5	0	0.0	22	27.8	
Under vehicle/train	0	0.0	0	0.0	0	0.0	0	0.0	0	0.0	2	2.5	
Fire/self-immolation	1	3.6	0	0.0	1	1.4	0	0.0	1	6.7	2	2.5	
Diving/drowning	0	0.0	2	2.0	1	1.4	0	0.0	0	0.0	1	1.3	
Unidentified	0	0.0	6	5.9	2	2.8	0	0.0	0	0.0	2	2.5	
**Place**													
Home	25	89.2	77	76.2	51	70.8	28	68.3	10	66.7	46	58.2	**< 0.001**
Work/school	1	3.6	4	4.0	3	4.2	3	7.3	0	0.0	8	10.1	
Worship/hospital	0	0.0	1	1.0	3	4.2	1	2.4	0	0.0	3	3.8	
Notable public sites	1	3.6	8	7.9	9	12.5	5	12.2	2	13.3	14	17.7	
Farm/forest/river	1	3.6	7	6.9	3	4.2	3	7.3	3	20.0	8	10.1	
Court/police cell	0	0.0	2	2.0	2	2.8	1	2.4	0	0.0	0	0.0	
Unidentified	0	0.0	2	2.0	1	1.4	0	0.0	0	0.0	0	0.0	
**Motivation**													
Relationship difficulties	17	60.7	30	29.7	17	23.6	4	9.8	2	13.3	12	15.2	**< 0.001**
Financial difficulties	0	0.0	7	6.9	15	20.8	14	34.1	3	20.0	16	20.3	
Poor academics	3	10.7	15	14.9	0	0.0	0	0.0	0	0.0	0	0.0	
Job difficulties	1	36	4	4.0	3	4.2	1	2.4	1	6.7	7	8.9	
Mental illness	2	7.1	23	22.8	11	15.3	7	17.1	2	13.3	13	16.5	
Loss events	1	3.6	5	5.0	11	15.3	5	12.2	0	0.0	2	2.5	
Illness/childlessness	0	0.0	1	1.0	3	4.2	2	4.9	2	13.3	4	5.1	
Multiple causes	0	0.0	0	0.0	2	2.8	0	0.0	0	0.0	0	0.0	
Unidentified	4	14.3	16	15.8	10	13.9	8	19.5	5	33.3	25	31.6	

*N*, total frequency; %, percentage; *p*, level of significance; bold, significant *p* value 0.05.

Unidentified, information not available in the reviewed media report.

### Age group differences on the type of suicidal behaviour, reported methods, places and motivation of suicidal behaviour

Poisoning was the most common method in people who were under 30 years of age, and hanging was the most common for people who were aged 30 years and above. Significant associations (*p* < 0.001) were observed between age groups and reported places where the suicidal behaviour took place. Home was the most common across the age groups, but its proportion of suicidal behaviour occurring at home decreased as the age groups increased. Differently, the proportion of suicidal behaviour that took place in notable public sites and farm, forest and/or river increased as the age groups increased.

There were significant differences (*p* < 0.001) between identified age groups and narratives of motivation. Relationship difficulties (60.7%) and poor academics (10.7%) were the most common in victims and/or survivors under 18 years of age, whereas relationship difficulties (29.7%) and mental disorders (22.8%) were the most common for the 18–29 years age group. Relationship difficulties (23.6%) and financial difficulties (20.8%) were the most common for the age group of 30–44 years, whilst for 45–59 years age group, financial difficulties (34.1%) and mental disorders (17.1%) were the most common. For victims and/or survivors of 60 years and above, financial difficulty (20.0%) was the most common. The rest of the results are presented in Table 4.

## Discussion

This study provides a situational analysis of patterns and trends of suicide in Nigeria through the analysis of media reports of 336 suicide events from 01 January 2016 to 31 December 2019. Completed suicide represented 78.9% of the reported suicidal behaviours, which is in contrast to estimates from the WHO which inferred that attempted suicides are often 20 times the number of completed suicides in the world.^2^

The difference may be because of the criminalisation of attempted suicide. According to Nigeria’s penal code, chapter 27, section 327, ‘Any person who attempts to kill himself is guilty of a misdemeanour, and is liable to imprisonment for 1 year’.^19^ Nigerian law also criminalises abetment of suicide. Also, under chapter 27, section 326 of the Nigeria penal code:

Any person who (1) procures another to kill himself; or (2) counsels another to kill himself and thereby induces him to do so; or (3) aids another in killing himself; is guilty of a felony, and is liable, to imprisonment for life.^22,23^

Therefore, it can be argued that information and news about suicide attempts are likely to be hidden and possibly underreported because of fear of prosecution by law-enforcement agencies. We advocate for the repeal of sections 326 and 327 of the Nigerian penal code that criminalise attempted suicide and its abetment.

### Age and suicidal behaviour

The frequency of suicidal behaviour rate was higher amongst 18–29 years age group, and thereafter it decreased with age. Other studies that were performed in low- and middle-income countries reported similar findings.^24,25,26^ However, suicide rates have been found to increase with age in most western and developed nations.^27,28^ The difference may be because of life expectancy which is lower in most low- and middle-income countries.^29^ Several factors, such as difficulty to cope with adversity or social problems, limited resources and network, may be contributing to higher rates of suicidal behaviour amongst young people, unlike older adults who probably have a stable network of resources.^25^ Also, young adults are more prone to substance abuse disorders and mood disorders which are the risk factors of suicide.^25,26,30,31,32^

### Gender and suicidal behaviour

Female suicidal behaviour rate was higher than the male suicidal behaviour rate amongst the sub-population of less than 18 years. All of the women below 18 years reported relationship problems, such as breakup initiated by their boyfriends because of unwanted pregnancies and parental disapproval of their relationships, as either their assigned or their confessed reasons for the suicide events. This suggests that suicidal behaviour could be a psychological complication of adolescent pregnancy.^33^ Furthermore, the sociocultural authority and position of parents that underpin parental disapproval of adolescent relationships and friendships appear to increase the risks of suicidal behaviour amongst adolescents.^34^ To halt this, parents and guardians should demonstrate respect for the views of their adolescent, even though adolescents have been raised by society to be obedient and to respect their parents and elders.^34^

### Method of suicidal behaviour

Hanging was the most common method amongst men, whereas poisoning was the most common method amongst women. This pattern can be explained by the differences in personality characteristics between men and women.^35,36^ For example, men mostly choose more violent means like hanging and firearm. For age groups, poisoning was the most common method in subjects below 30 years, whereas hanging was the most common amongst those that were 30 years and above. These results are similar to those of the WHO data, which report that, in most of the studied countries, hanging was the most frequent cause of suicide amongst men, and that there is an emerging trend of poisoning with pesticides in low- and middle-income countries because of the ease of access.^2,37,38^ In the context of Nigeria, pesticides are sold by street vendors and petty-traders for use in killing rodents and mosquitoes in many homes.^39^

### Motivation for suicidal behaviour

Relationship difficulties were the most common in subjects less than 45 years, whereas financial difficulties were the most common amongst those that were 45 years and above. This pattern underscores the peculiar challenges of the different groups, that is, one group is faced with finding a trustworthy partner and the other group is faced having to take care of dependents. The possible explanations could be that relationship and financial difficulties could trigger suicidal behaviour because of the feelings of being humiliated, entrapped, defeated and burdensomeness.^40^ Lastly, mental disorders could trigger the feelings of burdensomeness secondary to impairment from the illness and thwarted belongingness.^32^ Moreover, psychiatric illnesses are the major risk factors of suicide.^32^

### Limitations

Our findings only reflect the suicidal events captured by the online news media and are, therefore, not the actual estimates of suicidal events in Nigeria. However, in the absence of a comprehensive official source on suicide deaths in Nigeria, perhaps the only available sources of information are media articles on suicide.

Also, the study methodology did not look at the protective factors of suicidal behaviour amongst the reported suicide events because the available information from the media articles did not include these factors. Despite these limitations, this study provides valuable insights into the trends and patterns of suicidal behaviour in Nigeria.

### Recommendations

Pertinently, the information from this study should serve as an empirical basis for suicide risk reduction and prevention strategies, such as having a national guideline on media reporting of suicidal behaviour, establishing a national suicide registry, decriminalisation of suicidal behaviour and restriction of access to poisons.

## Conclusion

This study confirms that there are gender and age differences in the trends and patterns of suicidal behaviour in Nigeria. The study results buttress the need for a suicide prevention strategy in Nigeria. If successfully formed, risk reduction programmes, such as target interventions to vulnerable groups such as young adults and female adolescents, and restriction of access to pesticides, can be implemented. There is no better way to do this than the passage of the mental health bill in Nigeria, which will provide a framework for these target interventions, and increase access to care for vulnerable people.

## References

[1] World Health Organization. Preventing suicide: A global imperative [homepage on the Internet]. WHO; 2014 [cited 2020 Mar 25]. Available from: http://apps.who.int/iris/bitstream/10665/131056/1/9789241564779_eng.pdf

[2] World Health Organization. Suicide prevention [homepage on the Internet]. WHO; 2016 [cited 2020 Mar 25]. Available from: http://www.who.int/mental_health/suicide-prevention/en/

[3] Nnorom K. Social anomie and suicide phenomenon in Nigeria: Lending credence to the voiceless phenomenon. Glob J Hum-Soc Sci Res [serial online]. 2019 [cited 2020 Mar 24]. Available from: https://socialscienceresearch.org/index.php/GJHSS/article/view/3019

[4] Asuni T. Suicide in Western Nigeria. Br Med J. 1962;2(5312):1091–1097. 10.1136/bmj.2.5312.1091 13965316 PMC1926472

[5] Eferakeya AE. Drugs and suicide attempts in Benin city, Nigeria. Br J Psychiatry. 1984;145(1):70–73. 10.1192/bjp.145.1.70 6743947

[6] Gureje O, Kola L, Uwakwe R, Udofia O, Wakil A, Afolabi E. The profile and risks of suicidal behaviours in the Nigerian survey of mental health and well-being. Psychol Med. 2007;37(6):821–830. 10.1017/S0033291707000311 17349104

[7] Uwakwe R, Gureje O. The relationship of comorbidity of mental and substance use disorders with suicidal behaviours in the Nigerian survey of mental health and wellbeing. Soc Psychiatry Psychiatr Epidemiol. 2011;46(3):173–180. 10.1007/s00127-009-0178-2 20135089

[8] Adewuya AO, Ola BA, Coker OA, et al. Prevalence and associated factors for suicidal ideation in the Lagos State mental health survey, Nigeria. Br J Psych Open. 2016;2(6):385–389. 10.1192/bjpo.bp.116.004333 PMC5153566 27990294

[9] Armstrong G, Vijayakumar L, Niederkrotenthaler T, et al. Assessing the quality of media reporting of suicide news in India against World Health Organization guidelines: A content analysis study of nine major newspapers in Tamil Nadu. Aust N Z J Psychiatry. 2018;52(9):856–863. 10.1177/0004867418772343 29726275

[10] Akinyemi A, Isiugo-Abanihe U. Demographic dynamics and development in Nigeria. Afr Pop Stud. 2014;27(2):239–248. 10.11564/27-2-471

[11] Nigeria profile – Media [homepage on the Internet]. [cited 2020 Mar 25]. Available from: https://www.bbc.com/news/world-africa-13949549

[12] The Nigeria media: Evolution, trends and projections for 2018 – Part 1 [homepage on the Internet]. [cited 2020 Mar 25]. Available from: https://guardian.ng/features/the-nigeria-media-evolution-trends-and-projections-for-2018-part-1/

[13] Sisask M, Värnik A. Media roles in suicide prevention: A systematic review. Int J Environ Res Public Health. 2012;9(1):123–138. 10.3390/ijerph9010123 22470283 PMC3315075

[14] Zalsman G, Hawton K, Wasserman D, et al. Suicide prevention strategies revisited: 10-year systematic review. Lancet Psychiatry. 2016;3(7):646–659. 10.1016/S2215-0366(16)30030-X 27289303

[15] Lee J, Lee WY, Hwang JS, Stack SJ. To what extent does the reporting behavior of the media regarding a celebrity suicide influence subsequent suicides in South Korea? Suicide Life-Threat Behav. 2014;44(4):457–472. 10.1111/sltb.12109 25041623

[16] Niederkrotenthaler T, Fu KW, Yip PS, et al. Changes in suicide rates following media reports on celebrity suicide: A meta-analysis. J Epidemiol Community Health. 2012;66(11):1037–1042. 10.1136/jech-2011-200707 22523342

[17] Brown KM, Elliott SJ, Leatherdale ST, Robertson-Wilson J. Searching for rigour in the reporting of mixed methods population health research: A methodological review. Health Educ Res. 2015;30(6):811–839. 10.1093/her/cyv046.Epub 26491072

[18] Cheng AT, Hawton K, Chen TH, et al. The influence of media reporting of a celebrity suicide on suicidal behavior in patients with a history of depressive disorder. J Affect Disord. 2007;103(1–3):69–75. 10.1016/j.jad.2007.01.021 17313978

[19] Top sites in Nigeria [homepage on the Internet]. [cited 2020 Jan 5]. Available from: https://www.alexa.com/topsites/countries/NG

[20] Moher D, Liberati A, Tetzlaff J, Altman DG, PRISMA Group. Preferred reporting items for systematic reviews and meta-analyses: The PRISMA statement. Br Med J. 2009;339:b2535. 10.1136/bmj.b2535 21603045 PMC3090117

[21] IBM Corp. Released 2015. IBM SPSS statistics for Windows, version 23.0. Armonk, NY: IBM Corp; 2015.

[22] Nigeria: Criminal Code Act [Nigeria]. Cap C38 LFN 2004 [homepage on the Internet]. [cited 2020 Dec 25]. Available from: https://www.refworld.org/docid/49997ade1a.html

[23] Adinkrah M. Anti-suicide laws in nine African countries: Criminalization, prosecution and penalization. Afr J Criminol Justice Stud. 2016;9:279–292.

[24] Reddy MS. Suicide incidence and epidemiology. Indian J Psychol Med. 2010;32(2):77–82. 10.4103/0253-7176.78501 21716862 PMC3122543

[25] Abdulai T. Trends of online news media reported suicides in Ghana (1997–2019). BMC Public Health. 2020;20(1):35. 10.1186/s12889-020-8149-3 31918688 PMC6953180

[26] Turecki G. Suicide and suicidal behaviour. Lancet. 2017;387(10024):1227–1239. 10.1016/S0140-6736(15)00234-2 PMC5319859 26385066

[27] Barak Y, Cheung G, Fortune S, Glue P. No country for older men: ageing male suicide in New Zealand. Australas Psychiatry. 2020;28(4):383–385. 10.1177/1039856220905304 32093500

[28] Kim JW, Jung HY, Won DY, Shin YS, Noh JH, Kang TI. Landscape of elderly suicide in South Korea: Its trend according to age, gender, and educational attainment. Omega (Westport). 2020;82(2):214–229. 10.1177/0030222818807845 30360680

[29] WHO. World Health Statistics (Monitoring health for the SDGs). Geneva: World Health Organization (WHO), 2016; p. 2016.

[30] Dzamalala CP, Milner DA, Liomba NG. Suicide in Blantyre, Malawi (2000–2003). J Clin Forensic Med. 2006;13(2):65–69. 10.1016/j.jcfm.2005.08.006 16271492

[31] Mars B, Burrows S, Hjelmeland H, Gunnell D. Suicidal behaviour across the African continent: A review of the literature. BMC Public Health. 2014;14:606. 10.1186/1471-2458-14-606 24927746 PMC4067111

[32] Adinkrah M. Epidemiologic characteristics of suicidal behavior in contemporary Ghana. Crisis. 2011;32(1):31–36. 10.1027/0227-5910/a000056 21371968

[33] Musyimi CW, Mutiso VN, Nyamai DN, Ebuenyi I, Ndetei DM. Suicidal behavior risks during adolescent pregnancy in a low-resource setting: A qualitative study. PLoS One. 2020;15(7):e0236269. 10.1371/journal.pone.0236269 32697791 PMC7375578

[34] Quarshie EN, Osafo J, Akotia CS, Peprah J. Adolescent suicide in Ghana: A content analysis of media reports. Int J Qual Stud Health Well-being. 2015;10(1):27682. 10.3402/qhw.v10.27682 26015405 PMC4444762

[35] Callanan VJ, Davis MS. Gender differences in suicide methods. Soc Psychiatry Psychiatr Epidemiol. 2012;47(6):857–869. 10.1007/s00127-011-0393-5 21604180

[36] McAndrew FT, Garrison AJ. Beliefs about gender differences in methods and causes of suicide. Arch Suicide Res. 2007;11(3):271–279. 10.1080/13811110701403940 17558612

[37] Jørs E, Neupane D, London L. Pesticide poisonings in low- and middle-income countries. Environ Health Insights. 2018;12. 10.1177/1178630217750876 PMC5757432 29326530

[38] Gunnell D, Eddleston M, Phillips MR, Konradsen F. The global distribution of fatal pesticide self-poisoning: Systematic review. BMC Public Health. 2007;7:357 10.1186/1471-2458-7-357 18154668 PMC2262093

[39] Traders in Abuja freely sell snipers in open markets, month after NAFDAC banned product [homepage on the Internet]. [cited 2020 Mar 25]. Available from: https://www.icirnigeria.org/

[40] Adinkrah M. Intimate partner femicide – Suicides in Ghana: Victims, offenders, and incident characteristics. Violence against Wom. 2014;20(9):1079–1096. 10.1177/1077801214549637 25261436

